# Galectin-3—Insights from Inflammatory Bowel Disease and Primary Sclerosing Cholangitis

**DOI:** 10.3390/ijms26136101

**Published:** 2025-06-25

**Authors:** Thomas Grewal, Hauke Christian Tews, Christa Buechler

**Affiliations:** 1 School of Pharmacy, Faculty of Medicine and Health, University of Sydney, Sydney, NSW 2006, Australia; thomas.grewal@sydney.edu.au; 2 Department of Internal Medicine I, University Hospital Regensburg, 93053 Regensburg, Germany; hauke.tews@klinik.uni-regensburg.de

**Keywords:** inflammatory bowel disease, primary sclerosing cholangitis, galectin-3

## Abstract

Inflammatory bowel disease (IBD) and primary sclerosing cholangitis (PSC) are related diseases with poorly understood pathophysiology. While therapy options for IBD have increased, treatment options for PSC remain limited. Galectin-3 is a multifunctional lectin expressed in intestinal epithelial cells, and is abundant in immune cells such as macrophages, with roles in cell adhesion, apoptosis, inflammation and fibrosis being associated with IBD and PSC disease development and progression. In addition, galectin-3 is also a visceral fat-derived protein whose systemic levels are increased in obese individuals, the latter correlating with a poorer prognosis in IBD and PSC patients. On the other hand, decreased galectin-3 expression in the inflamed mucosal tissues of mice and patients with IBD possibly indicate a protective role of this lectin in IBD. However, galectin-3 loss or inhibition is protective in most animal models of liver fibrosis but exacerbates the severity of autoimmune liver disease. Hence, with PSC being a slowly progressing autoimmune hepatobiliary disease closely related to IBD, further studies evaluating galectin-3 as a therapeutic target or biomarker for the severity of IBD and the occurrence of PSC are still needed. This review summarizes studies that have analyzed expression patterns and functions of galectin-3 in IBD and PSC. Current evidence suggests that strategies to block galectin-3 are not advised for patients with IBD and PSC-IBD.

## 1. Introduction

Inflammatory bowel disease (IBD), which includes Crohn’s disease (CD) and ulcerative colitis (UC), is a chronic inflammatory disorder characterized by intestinal barrier dysfunction that is becoming increasingly prevalent and common, particularly in the developed world [1,2,3,4,5,6]. Although considerable research has identified genetic predispositions, microbial imbalances, immune system irregularities and environmental factors as potential contributors to increased intestinal permeability during the development of IBD, the etiology of these diseases remains elusive [7]. IBD is a complex disorder involving the dysregulation of the mucosal immune system, submucosal layers and factors impacting on luminal barrier function and immune responses, such as luminal contents of the intestinal tract, including the gut microbiota, bile acids and macronutrients from the diet [8,9,10,11,12].

While the inflammation in UC is limited to the large bowel, CD can affect any part of the gastrointestinal tract [13,14]. To effectively diagnose CD and UC, contemporary medical technologies, including endoscopy, colonoscopy and upper gastrointestinal radiography have been instrumental in distinguishing between the two conditions. Nevertheless, diagnosing these diseases can be particularly challenging in cases of severe illness and significant colon infection [15]. Ongoing studies continuously aim to identify biomarkers for the differentiation of patients with CD and UC [16].

In the case of IBD, fecal biomarkers play an important role in the diagnosis and monitoring of treatment response. Fecal calprotectin, a well-established marker for assessing intestinal inflammation and predicting disease flares in clinical practice [17,18], is released by granulocytes and elevated in various inflammatory conditions, making it non-specific to IBD [19,20]. Alternatively, S100A12 (calgranulin) and lipocalin-2 in serum and feces may also emerge as biomarkers for the diagnosis of a severely inflamed digestive tract in IBD (also termed ‘active’ IBD) [16].

The chronic inflammation associated with IBD can trigger fibrosis, characterized by increased synthesis and degradation of the extracellular matrix (ECM). Intestinal fibrosis is a common complication associated with IBD and contributes to intestinal strictures and fistulas, characterized by the accumulation of ECM proteins such as collagens [21]. The latter may provide an opportunity to develop biomarkers that reflect fibro-inflammatory activity in patients with IBD. This could possibly allow for the evaluation of disease stage and progression [22]. On this note, serum biomarkers related to collagen degradation have been described in IBD, discriminating between moderate and severe disease stages [23,24]. Hydroxyproline is abundant in collagen and is increased in fibrotic tissues and serum and thus has long been used to quantify fibrosis [25]. Collagens account for about one-third of the total protein content in the body, and since hydroxyproline is not used in protein synthesis, it is excreted by the liver and kidneys [26]. Indeed, urinary hydroxyproline levels were higher in patients with IBD compared to healthy controls, but did not correlate with disease severity, which was assessed by fecal calprotectin levels [27].

Primary sclerosing cholangitis (PSC) is a rare chronic biliary disease affecting less than 5 out of 10,000 individuals [28], and is commonly associated with IBD, with approximately 70% of patients diagnosed with PSC also being affected by IBD [29]. This progressive disease is characterized by the deterioration of the bile ducts, leading to cholestasis, liver fibrosis, and ultimately cirrhosis. There are currently no effective pharmaceutical interventions to halt the progression of the disease. Ursodeoxycholic acid at low doses can improve laboratory measures of liver disease severity, but its use is still controversial [30]. Balloon dilatation with and without stenting of the strictures can improve cholestasis [31]. Patients with IBD are recommended to undergo annual liver function tests, regardless of symptoms, for PSC [32]. The primary non-invasive diagnostic tool for PSC is magnetic resonance cholangiopancreatography [32,33]. However, this imaging technique is costly and can present challenges in accurately assessing the bile ducts, highlighting the need for non-invasive biomarkers [29].

Potential biomarker candidates include members of the galectin family, a distinct class of lectins that specifically bind to β-galactoside-containing ligands [34,35]. Galectin-3 (also known as Mac-2) is unique amongst galectins due to its single carbohydrate recognition domain (CRD) at the C-terminus. Located upstream are the intermediate domain, containing proline–glycine–alanine–tyrosine repeats, and a short N-terminal domain [35]. Galectin-3 can self-associate into oligomers via its N- or C-terminal domain. The binding of galectin-3 to its ligands generally involves the CRD [35], and its cooperative activity with a wide range of intra- and extracellular proteins mediates the interaction of cells with ECM proteins [36]. Galectin-3 binding to membrane lipids, such as glycosphingolipids or glycosylated cargo, leads to plasma membrane bending and biogenesis of carriers for clathrin-independent endocytosis [37]. Because of the numerous functions and wide expression patterns, e.g., by immune cells, adipocytes, epithelial and endothelial cells [38,39,40], galectin-3 has been implicated in numerous biological and pathological processes [41,42,43,44,45,46]. Overall, the main functions of galectin-3 could be summarized as (i) facilitating the initiation of acute inflammation and perpetuation of chronic inflammatory processes, and (ii) enabling fibroblast to myofibroblast transition as well as mesenchymal transition of endo- and epithelial cells. Hence, galectin-3 can be viewed as a proinflammatory protein that is capable of triggering fibrogenesis [35,44,47,48].

This review discusses recent advances in the understanding of galectin-3-related functions and molecular mechanisms in the context of IBD and IBD-associated PSC. In addition, we summarize studies that analyzed galectin-3 expression as a diagnostic and prognostic biomarker tool in patients with an inflamed gut.

## 2. IBD, Obesity and Galectin-3

Obesity is a continuously growing health issue in the general population and also affects IBD. Cross-sectional studies identified up to 40% of adult IBD patients to be overweight [49], which, together with obesity, has a global prevalence of nearly 40% and is considered a major health concern worldwide [50]. While increased IBD disease severity or the advancement of IBD-related complications do not consistently correlate with the prevalence of general obesity, an elevated risk of complications related to IBD is independently associated with visceral fat accumulation [49]. A study found a negative correlation between the time to the onset of an IBD flare and visceral adiposity, as opposed to body mass index [51]. Furthermore, a poorer response to medical therapy was associated with a higher intraabdominal visceral adipose tissue mass in patients with IBD [52].

These observations indicate a multifaceted relationship between obesity and IBD disease progression and severity. Further adding complexity, creeping fat is defined as an extraintestinal manifestation of IBD that develops in CD. This condition is characterized by the accumulation of mesenteric adipose tissue that encircles the intestinal wall. This fat depot has a unique secretome with a higher secretion of pro- as well as anti-inflammatory adipokines [53]. Creeping fat functions as a protective barrier against bacterial invasion [54] but can also compromise adjacent tissues and participate in disease complications [55].

Obesity and especially the accumulation of visceral adipose tissue have long been known to exacerbate the severity of chronic liver diseases [56,57,58,59]. In fact, visceral adiposity and its associated metabolic diseases, such as type 2 diabetes, are risk factors for metabolic dysfunction-associated fatty liver disease (MAFLD) [56,60,61]. The higher prevalence and progression of MAFLD in patients with IBD is likely a cause of chronic inflammation [62]. Further indicating a yet-to-be-clarified cooperativity, overweight, and especially obese, patients with PSC have a more rapid progression of liver fibrosis [63].

The molecular players that link IBD and obesity have yet to be identified, but as outlined in more detail below, the roles for galectin-3 in intestinal epithelium and adipose tissue may provide functional connectivity between these tissues.

### Galectin-3 and Obesity-Associated Disease

**Figure 1:**
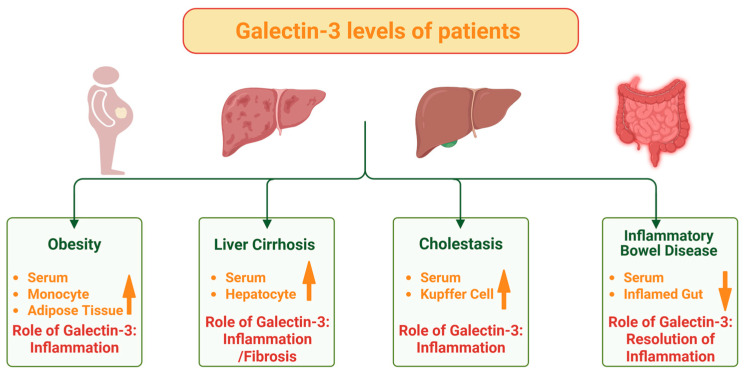
The expression and roles of galectin-3 in obesity, chronic liver diseases, and inflammatory bowel disease. (i) In obesity, galectin-3 is increased (↑) in the serum, monocytes and intraabdominal adipose tissues, with roles for galectin-3 in inflammation and insulin resistance. (ii) In liver cirrhosis, hepatocyte and serum galectin-3 levels are increased, and here galectin-3 has a role in inflammation and liver fibrosis. (iii) In cholestatic liver disease, galectin-3 levels in serum and Kupffer cells are increased, and here galectin-3 has a role in inflammation and liver fibrosis. (iv) In active Crohn’s disease, serum and gut expression of galectin-3 are mostly reduced (↓), and here galectin-3 has a role in the resolution of the inflammation. In patients with ulcerative colitis the serum levels and tissue expression of galectin-3 appear normal. Created in BioRender. Pollinger, K. (2025). https://BioRender.com/5m1y3np, accessed on 21 June 2025.

In CD patients, intraabdominal adipose tissue mass appears higher than in healthy controls, and together with other evidence, this implicates a link to more severe forms of the disease and higher recurrence rates after surgery [64]. Given increased galectin-3 expression levels in adipose tissues and circulating monocytes as well as elevated galectin-3 serum levels in the obese [39,65,66] (Figure 1), a heightened interest in the pathological functions of galectin-3 in obesity-associated diseases, such as type 2 diabetes and MAFLD, has developed in recent years [67,68].

Galectin-3 null mice are protected from obesity in a dietary model [69], and higher galectin-3 levels in the obese appear to contribute to metabolic disease. However, contesting these findings, others observed that galectin-3-deficient mice became more obese and displayed higher grades of adipose tissue inflammation. When challenged with lipopolysaccharide (LPS), activation of nuclear factor-κB (p65) in peritoneal macrophages of these animals increased [70]. The underlying cause for these rather opposite outcomes has yet to be clarified, as sex, genetic background, and dietary conditions in these studies were comparable [69,70].

Nevertheless, a dose-dependent role for galectin-3 in obesity was supported in galectin-3 null mice that were fed a high-fat diet for a prolonged time (6 months) to become more obese, developing excess metabolic disease compared to controls [71]. Yet, to further complicate matters, wild-type and galectin-3 null mice fed a high-fat, atherogenic diet for a similar time frame (8 months) displayed comparable body weights. Interestingly, the galectin-3-deficient mice were characterized by accelerated atherogenesis, partly caused by inefficient removal of atherogenic lipoproteins [72]. However, the latter observation is not supported by studies analyzing mice deficient in both apolipoprotein E and galectin-3, which exhibited normal serum lipid profiles and less atherosclerosis than the wild-type animals [73].

Although the role of galectin-3 in obesity is unclear from the above studies, there is convincing evidence that galectin-3 serum levels are increased in obesity and obesity-associated metabolic diseases [67]. Visceral adipose tissues produce higher levels of galectin-3 than subcutaneous fat, which is transported to the liver via the portal vein and may expose the liver to comparatively high levels of galectin-3 [65,74]. Therefore, galectin-3 may play a role in the adverse effects of visceral obesity in PSC and IBD, a hypothesis requiring further study.

## 3. Galectin-3 Regulates Intestinal Epithelial Cell Junctions

Intestinal epithelial cells play a key role in maintaining gut health and crucially contribute to IBD. The intestinal epithelium is a boundary between the internal and external environments of the body, which directly interacts with commensal and pathogenic bacteria, as well as environmental elements such as food [75]. The gut microbiome has a critical role in the digestion and absorption of incoming nutrients, immune functions, and overall metabolism [76,77,78]. Vice versa, diet significantly influences the composition of the gut microbiome [76,77,78]. Pointing at a role for galectin-3 at this interface of internal and external factors, galectin-3 null mice exhibited a different gut microbiome than controls in a model of nonsteroidal anti-inflammatory drug-induced ulcers [79]. Possibly transferring this observation into disease-like settings, to our knowledge, the microbiome of galectin-3 deficient mice with colitis has not yet been analyzed.

Increased permeability of the intestinal barrier critically contributes to IBD pathogenesis. The compromised functionality of the intestinal barrier is related to the breakdown of epithelial junctions [80]. These epithelial or adherens junctions mediate adhesion by providing cell–cell or cell–matrix contacts. They are composed of the transmembrane protein epithelial (E)-cadherin. Inside cells, E-cadherin is linked via catenins to cellular actin filaments, thereby connecting the plasma membrane to the actin cytoskeleton. The extracellular domains of E-cadherin can interact and connect epithelial cells. Importantly, E-cadherin dysfunction can contribute to disrupting epithelial barrier integrity, and inflammatory cytokines induce the translocation of E-cadherin from the membrane to the cytoplasm, destabilizing the intestinal barrier and increasing paracellular permeability [81].

Tight junctions are another type of specialized region that enable neighboring cells to make contact. They consist of adhesion protein complexes composed of proteins such as claudin, zonula occludens, and occludin. Tight junctions regulate the passage of small molecules, peptides, and proteins [82]. Intriguingly, in addition to inflammatory molecules triggering E-cadherin mislocalization (see above), these cytokines can also change the expression of claudins and zona occludens and contribute to the disruption of these complexes in IBD [82].

Furthermore, besides adherens and tight junctions, desmosomes function as strong cell–cell contacts and comprise cadherins, desmogleins and desmocollins [83]. These structures are crucial for intestinal epithelial barrier integrity. Disruption of tight junction and/or adherens junction formation can lead to the unprovoked onset of intestinal inflammation [75]. This inflammatory response can lead to desmosomes becoming dysfunctional and significantly contributing to the progression of intestinal inflammation and IBD pathogenesis. The network and crosstalk of molecular events that link adherens junctions, tight junctions and desmosomes to control epithelial cell-to-cell adherence and epithelial barrier function remain to be fully understood, but changes in their ability to regulate the actin cytoskeleton as well as intracellular signaling pathways probably contribute to IBD onset and progression [81,82,83].

Disintegration of adhesion junctions, tight junctions, and desmosomes is commonly associated with a loss of cell polarity and is closely linked to the transdifferentiation of epithelial cells into mesenchymal cells, a phenomenon known as epithelial–mesenchymal transition [84]. During this process, epithelial cells acquire mesenchymal properties, which are essential for fibrosis [85]. The reactivation of epithelial–mesenchymal transition is generally considered to reflect a physiological response aimed at regulating inflammation and facilitating the repair of injured tissue [85]. Epithelial cells in the injured tissues of IBD patients acquire an inflammatory phenotype and have increased regenerative capacities [86], which may ultimately result in tissue fibrosis.

The ability of adherens/tight junctions and desmosomes to ensure epithelial barrier function could be influenced by galectin-3, as galectin-3 overexpression in NCM460 cells, a human colon mucosal epithelial cell line, impaired epithelial barrier function, and increased inflammation, and apoptosis [87]. Moreover, dextran sodium sulphate (DSS), which is used to induce colitis in animal models [88], elevated galectin-3 expression in these cells, indicating that this scenario could be relevant when intestinal barrier integrity is compromised.

**Figure 2:**
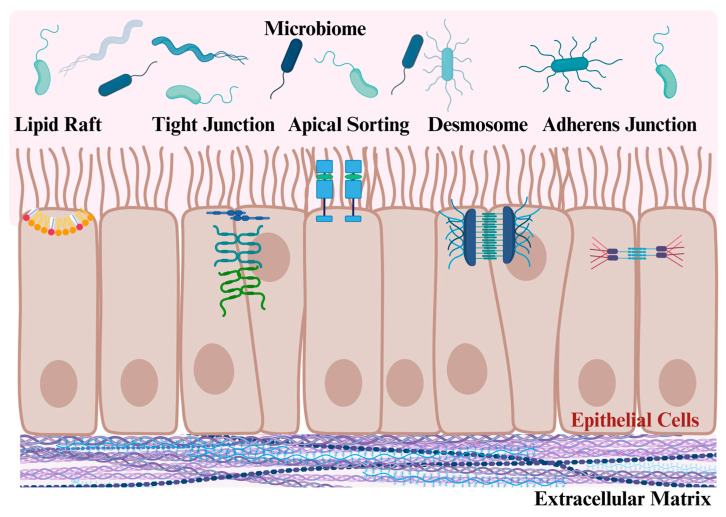
The multiple roles of galectin-3 influencing barrier integrity in epithelial cells. (i) Galectin-3 modifies lipid rafts, and this may alter signaling of lipid raft localized molecules. (ii) Tight junction, adherens junction and desmosome stability are regulated by galectin-3. (iii) Galectin-3 binds to extracellular matrix proteins, which may also affect epithelial barrier permeability. (iv) The apical sorting of proteins is regulated by galectin-3. (v) Galectin-3 has antimicrobial activities and might alter the gut microbiome. The pathophysiological relevance of these possible roles for galectin-3 in epithelial cells remains unclear, with galectin-3 null mice exhibiting rather normal epithelial histology in the intestinal tract. See text for further details. Created in BioRender. Pollinger, K. (2025). https://BioRender.com/d6xrwd1, accessed on 21 June 2025.

Current evidence from cell-based studies suggests that galectin-3 disrupts adherens and tight junctions while stabilizing desmosomes, which all affect the barrier function of epithelial cells (Figure 2) [84,89]. Several underlying mechanisms, involving varied galectin-3 interaction partners, appear relevant in this context. For instance, galectin-3 can form a complex with N-cadherin, which then accumulates in cell membrane rafts to destabilize adherens junctions [84,90]. In addition, using the cornea as a model, galectin-3 facilitated cell detachment and redistribution of the tight junction protein occludin via its N-terminal polymerizing domain [91]. In the human A549 airway epithelial cell line, recombinant galectin-3 induced secretion of matrix metalloproteinases, and their increased ability to degrade ECM led to the disruption of cell–cell tight junctions, and a significant increase in paracellular permeability [92].

Yet, not all studies point at a destabilizing function of galectin-3 for epithelial integrity. For example, in human SKCO-15 and T84 intestinal epithelial cell lines, the binding of galectin-3 to the extracellular domain of desmoglein 2 stabilized this transmembrane protein and consequently increased the intercellular adhesion of intestinal epithelial cells. In this model, downregulation of galectin-3 or the addition of lactose, a competitor of galectin-3 binding, lowered intercellular adhesion of these intestinal epithelial cell lines [89].

Introducing further intricacy, extracellular galectin-3 can bind to a plethora of glycosylated receptors at the cell surface, as well as ECM proteins, all of which with often opposite consequences for cell–cell and cell–matrix adhesions. This includes the binding of galectin-3 to cell surface proteins, such as laminin or integrins, which either inhibits or promotes adhesion (Figure 2) [84,89,90,92].

Furthermore, the membrane microdomain localization of many proteins linked to adhesion and epithelial integrity is essential for their function. In fact, a lot of these proteins are localized in specialized microdomains rich in cholesterol, commonly named lipid rafts. Along these lines, lipid rafts isolated from mice with mild colitis and UC patients with inactive disease were disrupted [93], the former showing lipid raft disorganization prior to the increase in intestinal permeability [93]. Galectin-3 localized in lipid rafts was associated with an increased migration of activated dendritic cells and macrophages [94]. The ability of galectin-3 to bind glycosphingolipids and glycoproteins at the cell surface could influence lipid raft formation and composition, and thereby affect signaling events emanating from lipid rafts, with consequences for cell function [95]. Moreover, galectin-3 was shown to have a role for the sorting of apical membrane proteins in polarized epithelial cells, with recombinant galectin-3 or galectin-3 overexpression mediating the apical localization of beta-1 integrin in epithelial cells, while galectin-3 depletion caused apical proteins to reside in the basolateral membrane (Figure 2) [96,97]. Beta-1 integrin is essential for cell adhesion, and a peptide that colocalized with and increased beta-1 integrin expression in epithelial cells reduced inflammation in the colons of mice with DSS-induced colitis [98]. In line with a sorting function at the apical side of epithelial cells, possibly involving direct protein and lipid interactions within raft domains, the vast majority of recombinant galectin-3 added to Madin–Darby canine kidney cells were associated with the apical membrane, while only <5% of galectin-3 was detected in the basolateral domain [97].

Taken together, the function of galectin-3 in epithelial barrier permeability, stability, and function is very complex. One can envisage that (patho-)physiological changes in the epithelial microenvironment and localized spatiotemporal variations in ligand availability may trigger differential interactions of galectin-3 with its ligands, thereby contributing to diverse outcomes. In vivo, the sum of the above-listed multiple galectin-3-driven activities probably determines the overall impact on epithelial integrity and physiological consequences. In fact, the small intestine of galectin-3 null mice had a normal morphology [79], indicating that the effects of galectin-3 depletion may only become evident in the inflamed intestine. Hence, the role of galectin-3 in intestinal barrier stability is currently still unclear.

## 4. Macrophage, Neutrophil and T-Cell Function Is Regulated by Galectin-3

**Figure 3:**
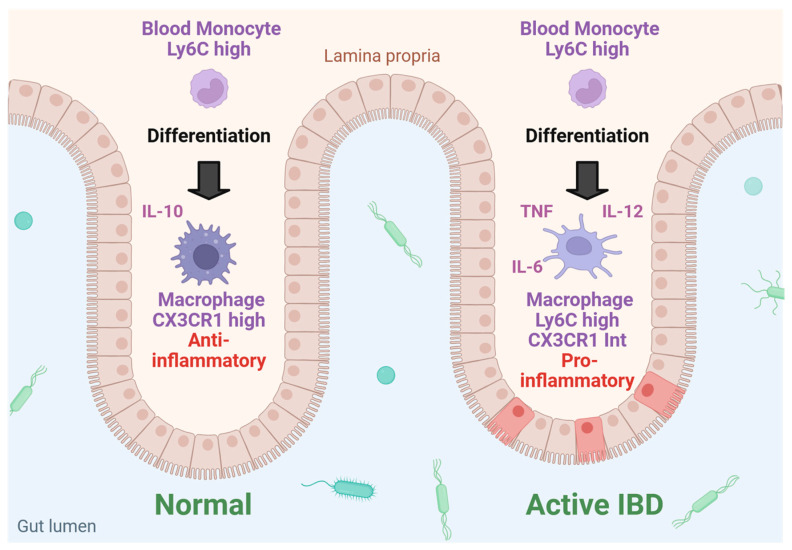
Intestinal macrophages in the normal (healthy) and inflamed gut. (i) In the normal gut, Ly6C^high^ monocytes from the blood differentiate to CX3CR1^high^ macrophages which produce IL-10 and exert anti-inflammatory activities. In contrast, in active inflammatory bowel disease (IBD), the Ly6C^high^ monocytes from the blood differentiate into CX3CR1^Int^ cells which produce TNF, IL-6 and IL-12 and exert proinflammatory activities. Created in BioRender. Pollinger, K. (2025). https://BioRender.com/3h1ygqi, accessed on 21 June 2025.

Macrophages have a central role in maintaining intestinal homeostasis and, when activated, promote IBD development and progression. Macrophages in the intestinal lamina propria, the thin layer of connective tissue underneath the intestinal epithelium that forms part of the mucosa, are constantly exposed to the content of the lumen, but are hypo-responsive to some stimuli, therefore exhibiting a reduced inflammatory response. This can in part be explained by their production of anti-inflammatory mediators, such as interleukin-10 (IL-10), limiting activation of the immune response [99] (Figure 3). Along these lines, even the removal of bacteria and cell debris by these cells was not associated with a proinflammatory response [100].

IL-10 is essential for the maintenance of intestinal homeostasis, and its anti-inflammatory properties efficiently overcome IBD in murine models. For instance, the beneficial effects of anti-tumor necrosis factor (TNF) therapy in IBD were due to increased IL-10 production from macrophages. T cells, which can also produce IL-10, were not critical for the therapeutic effects of this medication [101]. Strikingly, IL-10 null mice showed a spontaneous onset of gut inflammation. The histology of colitis in these mice was similar to that of human IBD, making the IL10-deficient mice a valuable experimental IBD model [102].

Given the protective role of intestinal macrophages for the epithelium and their potential to prevent inflammation associated with IBD, efforts have been made to better understand the recruitment process of macrophages into the intestinal mucosa. Depending on the expression levels of the marker protein lymphocyte antigen 6 complex (Ly6C), monocytes are classified as Ly6C^high^ and Ly6C^low^ [101]. Ly6C^high^ monocytes from the blood are recruited into the gut and differentiate into macrophages characterized by their specific expression of CX3C motif chemokine receptor 1 (CX3CR1) [99] (Figure 3). Recruitment of these cells to the intestine and colon is mediated by CC chemokine receptor type 2 which, interestingly, was not involved in the immigration of monocytes to the spleen or the liver [103]. Further ensuring the hypo-responsiveness to inflammatory stimuli, intestinal macrophages do not express receptors involved in innate immune response such as CD14, and do not release inflammatory cytokines when exposed to inflammatory agents [100].

However, under inflammatory conditions, as in colitis, Ly6C^high^ monocytes differentiate to proinflammatory macrophages and dendritic cells with high production of TNF, IL-6, IL-12 and IL-23 [104] (Figure 3). In addition, increased expression of monocyte toll-like receptor 2 and nucleotide-binding oligomerization domain 2 makes these cells more susceptible to inflammatory stimuli [99,104]. Ultimately, in the inflamed tissues, the majority of CX3CR1^high^ macrophages are replaced by CX3CR1^int^ cells (Figure 3), which also originate from Ly6C^high^ monocytes, but undergo a differentiation process different from the phagocytes in the non-inflamed tissues upon entry into the gut [103].

### The Multiple Roles of Galectin-3 in Inflamed Intestinal Epithelium

In addition to galectin-3 influencing barrier integrity in epithelial cells (see Section 3), galectin-3 also engages in multiple functions related to inflammation and the host response to bacteria and phagocytosis, some of which are summarized below. For more detailed descriptions, we refer the reader to excellent review articles [36,42,44,48].

Monocytes produce significant amounts of galectin-3, which further increase during phagocytic differentiation [105]. In relation to IBD, galectin-3 protein levels of intestinal macrophages of controls and patients with UC were similar, yet were low in intestinal macrophages of CD patients [106] (Table 1).

Several protective roles for galectin-3 contribute to host defense and immune response. This includes the ability of galectin-3 to recognize galactoside-containing glycoconjugates on pathogens like *Escherichia coli* or *Klebsiella pneumoniae* and thereby stimulate the host defense of phagocytes [48]. Galectin-3 also participates in the clearance of apoptotic immune cells and the activation of innate and adaptive immune cells such as macrophages, dendritic cells, natural killer cells, and T and B lymphocytes [107]. Mechanistically, galectin-3 promotes phagocytosis through both intracellular and extracellular processes. Inside cells, galectin-3 regulates the signaling pathways associated with the “eat me” signal, which involves the exposure of phosphatidylserine, followed by the activation of signaling cascades in phagocytes that enable the phagocytosis and clearance of apoptotic cells. In addition, extracellular galectin-3 acts as an opsonin, interacting with foreign intruders and pathogens, making them more recognizable to phagocytes [108].

When administered externally, galectin-3 enhances the respiratory burst in monocytes, acts as a chemoattractant for monocytes and macrophages, and increases their expression of CC chemokines [48,109]. In addition, extracellular galectin-3 affects the adhesion of immune cells to the ECM and facilitates apoptosis [110]. In contrast, endogenous galectin-3 acts as an anti-apoptotic agent [111,112]. The chemotaxis of J774 macrophages was enhanced by galectin-3 gene knockdown [113], and supplementation with exogenous galectin-3 was found to induce chemotaxis of these cells [114].

Although galectin-3 is considered to exert inflammatory effects, in resting and LPS-activated THP-1 macrophages the addition of exogenous recombinant galectin-3 did not increase the synthesis of IL-1beta, IL-6, IL-8, IL-10, and TNF, nor modulate its own production [115]. On the other hand, LPS and interferon-γ reduced galectin-3 levels in human monocytes [116]. This downregulation of galectin-3 levels in inflammatory settings, together with the, often opposing, roles of endogenous and exogenous galectin-3 highlights that a simple assignment of galectin-3 as a pro- or anti-inflammatory protein is not appropriate [36,42,44,48]. Galectin-3 has to be considered as an immunoregulatory protein which, probably due to the differential contribution of several modulating factors, may contribute to the initiation, but also the resolution of inflammation.

Macrophages from galectin-3 null mice showed an elevated response to LPS exposure, which was associated with increased inflammatory cytokine production compared to macrophages from control animals. Accordingly, galectin-3-deficient mice were more susceptible to LPS and displayed an excessive induction of inflammatory cytokines and nitric oxide production [117]. This enhanced inflammatory response was inhibited by the supplementation with recombinant galectin-3 [117]. In line with these findings, endogenous galectin-3 was found to exacerbate inflammation in a mouse model for asthma [118], while the pharmacological administration of galectin-3 revealed a protective effect in bronchial obstruction [119].

The passage of bacteria from the gastrointestinal tract to extraintestinal sites can contribute to local and systemic inflammation in IBD. However, despite its anti-inflammatory and opsonin features, it remains to be clarified whether galectin-3 can protect against bacterial overgrowth. To our knowledge, studies assessing intestinal macrophages for galectin-3 expression levels and their impact on inflammation are still lacking. Intestinal macrophages have diverse phenotypes and, as primary intestinal macrophages of patients and controls are not readily available, it remains a challenge to dissect and clarify the function of galectin-3 in these immune cells. Single-cell omics may be helpful to further elucidate the associations of galectin-3 and IBD [86].

Besides macrophages, several other cell types contribute to the development and progression of IBD. This includes dendritic cells, which present antigens to CD4 T cells. The latter then proliferate and differentiate into T helper 1 (Th1) and T helper 17 (Th17) cells, secreting interferon-γ and other proinflammatory cytokines such as IL-17 and IL-22, respectively [120]. In IBD, an overactivation of CD4 T cells secreting high levels of these proinflammatory mediators can substantially contribute to inflammation in the intestine [121]. This is substantiated by other studies that identified Th1, γδ T, and Th17 cells to contribute to inflammation in IBD [122]. This Th1/Th17 response is also evident in CD and UC. In addition, and compared to CD, UC is characterized by a Th2 response, which causes natural killer T cell expansion, further contributing to inflammatory disease progression [123].

The role of T cells in IBD development and advancement has been studied in further detail by introducing certain T cell populations into immunocompromised mice. As such, CD4^+^ CD45RB^high^ cells, Th1, Th17, and IL-10-deficient CD4^+^ T cells were transferred into immunodeficient mice to induce colitis [121]. Transfer of CD4^+^ CD45RB^low^ cells protected from CD4^+^ CD45RB^high^ cell-mediated colitis [121,124]. Transfer of regulatory T cells (CD4^+^ CD25^+^ CD45RB^low^) also improved inflammation in mice with colitis [121,125]. Alternatively, the expansion of regulatory T cells induced by the intestinal microbiome was shown to be protective in IBD [126].

Similar to the role of lipid rafts for the proper functioning of proteins linked to adhesion and epithelial integrity (see Section 3), T cells also contain a significant number of receptors and glycoproteins that are localized in these specialized and cholesterol-rich membrane microdomains in order to properly exert their functions. And like intestinal epithelial cells [94,95], galectin-3 also localizes to lipid rafts in T cells, affecting cell signaling and, consequently, T-cell function [127,128]. In particular, extracellular galectin-3 was found to alter membrane organization and prevent the translocation of the T cell receptor and CD4 into these membrane microdomains, thereby influencing T cell receptor signaling [127]. It was also described that galectin antagonists enhanced the cytotoxicity of CD8 tumor-infiltrating lymphocytes [129]. Altogether, current evidence implicates a predominant role for galectin-3 in the suppression of T cell activation, which could protect from autoreactive T cells in healthy settings. Along these lines, human CD4^+^CD25^high^ derived regulatory T cells, which control the immune response, downregulate immunostimulating molecules and limit autoinflammatory responses, and express high levels of galectin-3 protein [130]. On the other hand, galectin-3 would thereby also prevent an appropriate immune response to infection and inflammation [127].

Finally, a hallmark of active IBD and the innate immunity response is the infiltration of neutrophils in the intestinal mucosa. Neutrophils are rapidly recruited in response to bacteria and tissue injury. Proinflammatory cytokines secreted by phagocytosing macrophages stimulate neutrophils to release antimicrobial and cytotoxic proteins [131]. While this is part of a defense mechanism aiming to oppose pathogenic intruders, excessive or prolonged neutrophil activation can lead to chronic inflammation in IBD [132]. Further complicating matters, neutrophil function appears differentially affected in CD and UC, as neutrophil activation and proliferation have been implicated in UC, while defective neutrophils have been associated with CD [120]. Most relevant for disease management, neutrophils release calprotectin, which causes apoptosis and inhibits the proliferation of different cell types [133]. Moreover, fecal calprotectin has emerged as a valuable diagnostic tool to monitor gut inflammation in CD and UC, illustrating a central role for neutrophil activation in both disease entities [134].

Galectin-3 can also bind to neutrophils and thereby enhance the binding of these cells to laminin [110]. This activity of galectin-3 may contribute to the higher neutrophil infiltration in injured tissues [42]. Moreover, neutrophils opsonized with galectin-3 were more effectively cleared by macrophages [108], pointing at galectin-3 to initiate neutrophil recruitment in early inflammation and, at later stages, to resolve the inflammatory response.

Taken together, the above-listed diverse functions of galectin-3 in innate and adaptive immunity indicate an immunoregulatory rather than a solely inflammatory role of galectin-3 in IBD development and progression.

## 5. Galectin-3 in Experimental IBD

The composition of the gut microbiota of IBD patients differs from healthy individuals, with microbial diversity and the abundance of beneficial bacteria being reduced [19,76]. As outlined above, galectin-3 is an immunoregulatory and fibrotic protein that is expressed in intestinal epithelial cells [44,46,47,135], binds to bacterial products (e.g., LPS), acts as an opsonin for bacteria (e.g., *Candida* spp., *Helicobacter pylori*), and possesses antimicrobial activity [42]. These observations suggest that galectin-3 may play a role in immune tolerance to the gut microbiota [42].

Experimental studies have focused on the effect of galectin-3 in mouse models of colitis. DSS is a commonly used agent to induce IBD in rodents, as it compromises the epithelial barrier, thereby facilitating the invasion of intestinal bacteria into the mucosa [88]. This process leads to immune cell infiltration and subsequent inflammation. This increased permeability of the intestine (‘leaky gut’) can lead to inflammation of the liver and bile ducts, with the potential to progress to cirrhosis [88]. The combination of impaired liver function and intestinal inflammation contributes to intestinal dysbiosis, which can further exacerbate the disease [88,136].

Galectin-3 null mice and the respective wild-type controls served as models to address the role of galectin-3 in DSS-induced colitis. Despite the multiple functions of galectin-3 in epithelial cells (see Section 3), the small intestine of the galectin-3-deficient mice retains a normal morphology. The mucus level of goblet cells and mucus thickness at the luminal surface of the enterocytes from these animals were also comparable to controls [79].

In the wild-type mice, DSS caused epithelial cell damage, ulceration, extensive crypt loss, and significant immune cell infiltration. Strikingly, the galectin-3 knockout mice developed a much milder phenotype when given DSS [137]. In these mice, the production of proinflammatory cytokines and activation of the NOD-like receptor family, pyrin domain containing 3 (NLRP3) inflammasome in colonic macrophages, were reduced [137]. Further pointing to a crucial role of macrophage-derived galectin-3, the adoptive transfer of wild-type macrophages increased the disease severity of galectin-3 null mice [137]. Pectic polysaccharide reduced the colonic galectin-3 protein levels, inhibiting the interaction of galectin-3 with the NLRP3 complex and its ability to activate the NLRP3 inflammasome, thereby contributing to improving the colitis of DSS-treated mice [138].

**Figure 4:**
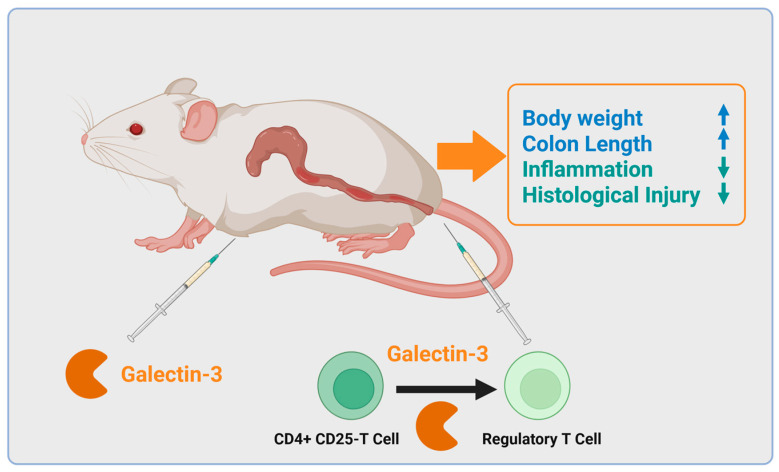
Galectin-3 protects from colitis. Intraperitoneal injection of galectin-3 improved DSS-induced colitis. Pretreatment of CD4^+^ CD25^−^ T cells with galectin-3 reduced their ability to trigger severe combined immunodeficiency and colitis as judged by higher body weight, longer colons, less intestinal inflammation and histological injury (see text for further details). Increased: ↑, Reduced: ↓. Created in BioRender. Pollinger, K. (2025). https://BioRender.com/uabg2j2, accessed on 21 June 2025.

Another study, also analyzing DSS-induced colitis in the galectin-3-deficient mouse model, suggested that galectin-3 is involved in the resolution of inflammation. In control animals, the initial strong proinflammatory response in DSS-induced disease ultimately enabled better recovery from colitis, which was not observed in galectin-3 knockout mice [139]. In this model, galectin-3 activated the Toll-like receptor 4 pathway in the dendritic cells of the colon, which subsequently induced regulatory T cells to suppress the Th1- and Th17-driven inflammation [139]. Genetic deletion of galectin-3 in mice thus resulted in impaired recovery from colitis with an increased number of Th1 and Th17 cells in the colon [139]. Galectin-3 treatment of T cells induced a regulatory T-cell phenotype, and adoptive transfer of these cells reduced colonic mucosa inflammation in galectin-3 null mice (Figure 4). Thus, in this model, galectin-3 inhibited colonic mucosa inflammation by inducing regulatory T cells [140].

Given that intra- and extracellular galectin-3 exerts different functions [67], the supplementation of recombinant galectin-3 in galectin-3 KO-mice strongly supported other findings on the function of exogenous galectin-3 in gut inflammation, providing a potential therapeutic approach to IBD. Notably, cellular uptake of extracellular galectin-3 also occurs, and internalization of recombinant galectin-3 by intestinal cells impacts cellular behavior needs further study [141].

Intraperitoneal injection of recombinant galectin-3 during DSS treatment in mice increased body weight (with low body weight being a marker of disease severity) and colonic length and improved mucosal inflammation [140] (Figure 4). In mice with severe combined immunodeficiency, where colitis was induced by the transfer of CD4^+^CD25^−^ positive T cells, pretreatment of these T cells with galectin-3 induced a phenotype resembling regulatory T cells, which suppressed inflammation, leading to less severe disease in this colitis model (Figure 4) [140].

In mouse models for chronic colitis, which involves four cycles of DSS treatment (each lasting 7 days), the intraperitoneal administration of recombinant galectin-3 for 5 days at 4 weeks after the induction phase led to a significant decrease in colonic IL-6 levels [142]. Further pointing to the protective role of galectin-3, its intraperitoneal delivery was associated with reduced weight loss and increased length of the colon in the acute DSS model (DSS for 7 days) (Figure 4) [142]. Follow-up studies then revealed that colonic lamina propria fibroblasts exposed to media from primary human colonic epithelial cells exhibited high IL-8 (CXCL8) production, which was ultimately attributed to galectin-3 being released by epithelial cells [143]. This indicates that the proinflammatory effects of galectin-3 may induce inflammation-resolving pathways triggered by IL-8 and essential for recovery from disease. In summary, although the relationship between dysbiosis, inappropriate immune response to gut bacteria, and galectin-3 needs further investigation, experimental models of colitis predominantly showed a protective role of galectin-3 in IBD.

## 6. Serum, Urinary and Fecal Galectin-3 in IBD

Taking into account the multitude of tasks and varied expression levels of galectin-3 in cells and tissues [38,39,40], both influencing the amounts of extracellular galectin-3 in a variety of pathological processes [41,42,43,44,45,46], efforts were made to correlate serum galectin-3 levels with disease. Indeed, systemic galectin-3 levels were found to be increased in type 2 diabetes, MAFLD, and infectious and autoimmune diseases and cancers [43] (Figure 1). Notably, a decline of galectin-3 levels was noticed in the sera of patients with rheumatoid arthritis [144]. Consequently, there were also studies analyzing galectin-3 amounts in the blood of patients with IBD.

LPS-incubated peripheral blood mononuclear cells from patients with UC produced higher amounts of galectin-3 compared to controls [137], which suggested elevated circulating galectin-3 levels in UC patients. Indeed, higher serum levels of galectin-3 in individuals with IBD have been reported, yet these upregulated amounts of systemic galectin-3 did not effectively differentiate between active disease and remission in patients with UC and CD [145,146] (Table 1). Hence, galectin-3 serum levels may not be a reliable biomarker for assessing disease activity in IBD. Experiencing additional difficulty in developing a common concept for galectin-3 levels in IBD, another study documented similar serum galectin-3 concentrations in IBD patients and healthy controls [147] (Table 1). Along these lines, recent work from our laboratories also showed comparable serum galectin-3 levels in controls and IBD patients (Table 1). In fact, serum galectin-3 amounts declined in patients with very severe IBD [148] (Figure 1). Furthermore, serum levels of galectin-3 in UC patients exhibited a negative correlation with both endoscopic and histological indicators of colitis (Table 1). In this cohort, increased fecal galectin-3 levels were identified as markers for remission in UC patients [139].

As the identification of new tools to detect disease continues to advance, it is becoming increasingly recognized that urinary proteins have significant biomarker potential for various pathologies [149]. Within this context, it was proposed that impaired renal excretion of galectin-3 could be responsible for its higher plasma concentrations [150]. Indeed, the presence of elevated galectin-3 levels in urine may indicate progressive kidney damage and renal fibrosis [149,151]. Furthermore, urine levels of galectin-3 have been recognized as a biomarker for heart failure and several types of cancer [149,152,153]. Renal complications occur in approximately 6% of IBD patients, with nephrolithiasis being a prevalent form [154,155]. This subgroup of IBD patients with renal complications tends to have a reduced glomerular filtration rate and elevated serum creatinine levels when compared to IBD patients with normal renal function [155].

Interestingly, low serum galectin-3 levels of IBD patients with active disease correlated with high galectin-3 amounts in urine [148]. The urinary protein/creatinine ratio as a measure of proteinuria and kidney dysfunction in these IBD patients [156] was normal [148]. The underlying mechanism for these observations remains unclear, but it appears unlikely that low serum galectin-3 levels of active IBD patients can be solely explained by higher renal excretion.

In summary, future studies analyzing systemic, fecal and urinary galectin-3 amounts will clarify if serum galectin-3 levels can be correlated with urinary and/or fecal excretion. It also remains to be evaluated if galectin-3 levels in the blood exert an effect on mucosal cells in IBD. Currently, overall evidence is inconclusive regarding systemic levels of galectin-3 and its correlation with disease activity in IBD. In fact, most currently available data point to the lack of significant differences in systemic galectin-3 levels when comparing controls with UC and CD patients [145,147,148].

## 7. Tissue Expression of Galectin-3 in IBD

As described above, IBD development and progression are influenced by systemic galectin-3 levels. Yet, the expression of galectin-3 in the gut may also contribute to IBD disease. Indeed, galectin-3 was highly expressed in the colon of UC patients, which positively correlated with histological and clinical scores [137] (Table 1). These findings and the positive correlation of colonic galectin-3 levels with macrophage markers suggested a role for macrophages in the induction of galectin-3 expression in UC [137]. In support of these studies, galectin-3 was also strongly expressed in the intestinal wall of colon specimens from UC patients. In this analysis, a trend towards lower galectin-3 expression in epithelial cells with higher grades of inflammation was observed, while immune cells displayed only minor galectin-3 amounts [157] (Table 1).

Inflammation of the ileal pouch, a surgical reservoir made from the ileum to store stool in patients that had undergone surgery with a partial or complete removal of both colon and rectum, is called pouchitis and is a common complication [158]. Interestingly, compared to patients without pouchitis, in subepithelial macrophages of UC patients with chronic and recurrent acute pouchitis, a significant reduction in the staining of galectin-3 was observed [159]. Pointing at tissue and cell-specific expression patterns of galectin-3 in different sections and cell types within the gut, galectin-3 expression in the lamina propria of small intestinal biopsies from the two patient groups was similar. Furthermore, galectin-3 staining was found in CD68^+^ macrophages but was not detectable in myofibroblasts [159]. It is tempting to speculate that these localized differences in expression patterns impact differentially on various galectin-3-dependent cellular activities.

Others observed low galectin-3 protein levels in epithelial cells in affected tissues of CD patients [160] (Table 1). Downregulation of galectin-3 in inflamed tissues of IBD patients was also noted in another study [161] (Table 1). The latter research showed TNF to suppress galectin-3 expression in normal biopsies and proposed low epithelial galectin-3 levels in inflamed mucosal tissue to reflect a typical immunological response [161]. Finally, when comparing colonic epithelial cells, colonic lamina propria fibroblasts, and intestinal macrophages from controls and IBD colonic tissue, significantly reduced galectin-3 amounts in the intestinal macrophages of CD patients were observed (Table 1). In the fistulae and stenosis of patients with CD, galectin-3 expression levels were only marginal [106]. Taken together, current analysis suggests increased colonic galectin-3 levels in UC patients, while CD appears associated with rather low galectin-3 expression in the affected tissues.

## 8. Galectin-3 in Liver Diseases and Primary Sclerosing Cholangitis

IBD is often found together with PSC, a chronic and slowly progressing liver disease where intra- and extrahepatic bile ducts become inflamed and scarred, ultimately leading to cholestasis, liver fibrosis, and cirrhosis. In this Section, we will therefore provide insights into the roles of galectin-3 in liver disease. Galectin-3 facilitates the activation of myofibroblasts [34,47], which in turn generate an excess of ECM, leading to the formation of scar tissue.

In a healthy liver, galectin-3 is detected in bile duct cells [74,162,163]. Elevated levels of galectin-3 in both hepatic tissue and serum have been observed in patients suffering from chronic liver diseases [47,74,164,165,166,167,168]. In cases of liver cirrhosis, there is a notable increase in the expression of galectin-3 in hepatocytes [34,47] (Figure 1). As galectin-3 activates myofibroblasts [34,47], higher expression of galectin-3 in the liver [47,167] may contribute to fibrogenesis (Figure 1).

In cholestasis, the expression of galectin-3 was primarily upregulated in Kupffer cells [162,163]. High levels of galectin-3 protein were also detected in biliary epithelial cells of patients with viral hepatitis, while galectin-3 was weakly expressed in biliary epithelial cells and sclerosing bile ducts of patients with primary biliary sclerosing cholangitis. On the other hand, the serum galectin-3 levels of these patients were higher compared to healthy controls [169] (Figure 1).

In support of a causative role in liver injury, studies involving galectin-3 knockout mice and those treated with galectin-3 inhibitors revealed a reduction in the number of liver-infiltrating mononuclear cells, which include B and T cells, dendritic and natural killer cells, and proinflammatory macrophages [170,171,172]. Furthermore, galectin-3 knockout mice and galectin-3 inhibitor-treated mice exhibited a notable increase in M2 macrophages, a subset of these cells typically being associated with anti-inflammatory responses [173]. Furthermore, mice lacking galectin-3 exhibited protection against liver fibrosis triggered by carbon tetrachloride treatment or non-alcoholic steatohepatitis [166,171] (Figure 1).

In a mouse model of autoimmune cholangitis leading to cholestatic liver injury, galectin-3 was found to play a crucial role in the activation of the macrophage inflammasome and the expression of IL-17, both of which are significant contributors to biliary epithelial damage [170,172]. Underscoring the notion that galectin-3 can exert divergent roles, galectin-3 knockout mice displayed more pronounced apoptosis of biliary epithelial cells when subjected to xenobiotic-induced cholestatic liver injury [169]. In these studies, the galectin-3-deficient mice were characterized by an increased inflammatory cell number, bile duct injury, and fibrosis (Figure 1). Hence, the impact of galectin-3 on the outcome in liver injury models may depend on the disease model, the specific cell types involved, and the differential contribution of intra- versus extracellular galectin-3 functions. Disruption of the galectin-3 gene in 129sv mice was consistent with the abovementioned findings, as it specifically inhibited IL-4/IL-13-induced alternative macrophage activation in bone marrow-derived macrophages in vitro, as well as in peritoneal macrophages in vivo, indicating a reduced ability to promote wound repair, the clearance of apoptotic cells, and resolution of inflammation [174].

The identification of non-invasive diagnostic and prognostic biomarkers for PSC remains an area of significant unmet need [175]. Current biomarkers, such as anti-neutrophil cytoplasmic antibodies, exhibit suboptimal efficacy [175]. Despite the varied effects of galectin-3 up- or downregulation in the mouse models described above, PSC patients displayed higher serum and urinary galectin-3 levels than IBD patients [148], suggesting that monitoring of galectin-3 amounts may be helpful for the early diagnosis of PSC in patients with IBD.

As noted earlier (see Section 6), higher plasma galectin-3 levels can be caused by impaired renal excretion [150]. However, in PSC patients, both serum and urinary galectin-3 levels were elevated. The urinary protein/creatinine ratio, as a measure of proteinuria [156], was normal, also showing that impaired renal galectin-3 excretion was not the main cause of higher serum galectin-3 levels.

In autoimmune immunoglobulin G4-related cholangitis, a systemic fibroinflammatory disorder that can affect various organs, with hepatobiliary manifestations being most common, galectin-3 autoantibodies were identified in a subset of patients. While the function of these autoantibodies against galectin-3 remains to be resolved, they were not detected in PSC patients [176], indicating that galectin-3 autoantibodies are not involved in PSC-related disease pathology.

Patients suffering from PSC with underlying IBD exhibit elevated serum levels of galectin-3 compared to those with isolated PSC [148]. As outlined above (see Section 5), galectin-3 may play a protective role in IBD [142,177], and the increased galectin-3 levels observed in PSC-IBD patients [148] could potentially contribute to the milder manifestations of IBD frequently noted in this group [29]. Therefore, extracellular galectin-3 in the serum may be protective or may enter the gut to exert beneficial functions. It is of particular interest that serum and urinary galectin-3 levels were not associated with elevated aminotransferase levels yet were clearly associated with PSC. Due to the limited patient number in this study [148], the potential of serum galectin-3 as a biomarker to distinguish between isolated PSC and PSC-IBD warrants further investigation.

## 9. Drugs Affecting Galectin-3 and Galectin-3 Antagonists for Liver Fibrosis

The differential impact of galectin-3 up- or downregulation on the functioning of the intestinal epithelium and immune response prompted studies investigating galectin-3 levels during treatment with drugs targeting IBD. Immunomodulatory drugs are widely used in IBD therapy [178] and were shown to regulate galectin-3 protein levels in THP-1 cells differentiated into macrophages using phorbol-12-myristate-13-acetate. Treatment with aspirin initially reduced galectin-3 protein levels, but these levels exceeded baseline levels after prolonged incubation. On the other hand, indomethacin, another nonsteroidal anti-inflammatory drug, transiently increased galectin-3 protein levels. Anti-inflammatory glucocorticoids such as hydrocortisone and dexamethasone also increased galectin-3 protein levels in THP-1 cells by a similar magnitude (20–40%) [179]. This illustrates that frequent treatment with glucocorticoid drugs, like cortisone in IBD, may induce galectin-3 expression in peripheral immune cells. It remains to be elucidated if galectin-3 upregulation confers some of the protective effects of these drugs in IBD therapy.

Metformin, a drug commonly used for the treatment of patients with type 2 diabetes, reduced galectin-3 levels in adipocytes and monocytes and correlated with reduced serum galectin-3 levels [65,66]. Prolonged metformin treatment (1 year) in IBD patients correlated with a lower probability for the need of intravenous steroids and reduced the need for IBD-related surgery in patients with CD [180]. In this retrospective cohort study, serum galectin-3 levels were not analyzed and whether or not metformin-related therapeutic benefits were mediated via changes in galectin-3 levels remains to be determined. In fact, other scenarios exist, as metformin increased glucose utilization and glucagon-like peptide-1 levels in the intestine [181]. IBD patients treated with glucagon-like peptide-based therapies showed reduced adverse outcomes in comparison to patients treated with different antidiabetics [182]. Moreover, as metformin changes the composition of the gut microbiome [181], other beneficial mechanisms of metformin for the treatment of IBD and unrelated to galectin-3 may exist.

The effect of several other medications on galectin-3 levels have been studied, but a potential link to IBD therapy is yet lacking. For instance, the sodium–glucose cotransporter 2 inhibitor empagliflozin, which is also used for the treatment of type 2 diabetes, did not change serum galectin-3 levels within 20 weeks of therapy [183]. Commonly used statins [184], such as rosuvastatin, but not atorvastatin, reduced galectin-3 levels in patients with acute myocardial infarction [185].

While a clear pathway to galectin-3 based IBD therapies has yet to be developed, the better understanding of the role of galectin-3 in fibrosis has prompted the development of galectin-3 antagonists as potential antifibrotic therapies. Both genetic and pharmacological inhibition of galectin-3 has led to improvements in renal dysfunction across different pathological scenarios [41]. Furthermore, experimental models of liver fibrosis have demonstrated the protective effects of galectin-3 [47,166,171]. Strong evidence for the fibrotic function of galectin-3 has made it a target for antifibrotic therapies [46]. Currently, the impact of galectin-3 inhibitors is under investigation in patients with liver fibrosis, but, thus far, the drugs evaluated have not demonstrated efficacy in improving fibrosis [46].

## 10. Conclusions

Galectin-3 is traditionally considered as an inflammatory and fibrotic protein (Figure 1). This view is greatly challenged in diseases with autoimmune characteristics, such as IBD, where galectin-3 exerts anti-inflammatory and beneficial effects. Based on the majority of studies, one can postulate that galectin-3 protects from inflammation and mucosal damage in IBD. This hypothesis is in agreement with the low expression levels of galectin-3 in the inflamed tissues of CD patients. However, serum galectin-3 amounts are not suitable as a biomarker for IBD diagnosis and disease monitoring. PSC-IBD is a chronic hepatobiliary disease with increased serum galectin-3 levels, a finding that needs confirmation in larger cohorts. In consideration of the protective effects of galectin-3 in IBD, galectin-3 antagonists, which are currently being tested as therapeutic options to improve renal and liver fibrosis, are not recommended for patients with PSC-IBD.

## Figures and Tables

**Table 1:** Serum levels and tissue expression of galectin-3 in patients with Crohn’s disease or ulcerative colitis. Please see test for details and references.

	Crohn’s Disease	Ulcerative Colitis
Serum Galectin-3	Increased	Increased
Serum Galectin-3		Reduced
Serum Galectin-3	Increased	Increased
Serum Galectin-3	Normal	Normal
Serum Galectin-3	Normal	Normal
Colonic tissue	Reduced	Normal
Intestinal macrophages	Reduced	Normal
Colonic tissue		Trend to lower levels
Intestinal epithelial cells	Reduced	
Colonic tissue		Increased
Endoscopic biopsies	Reduced in IBD	

## Data Availability

Not applicable.

## Abbreviations

The following abbreviations are used in this manuscript:

CD	Crohn’s disease
CRD	Carbohydrate recognition domain
CX3CR1	CX3C motif chemokine receptor 1
E-cadherin	Epithelial cadherin
ECM	Extracellular matrix
DSS	Dextran sodium sulfate
IBD	Inflammatory bowel disease
IL	Interleukin
LPS	Lipopolysaccharide
Ly6C	Lymphocyte antigen 6 complex
MAFLD	Metabolic dysfunction-associated fatty liver disease
NLRP3	NOD-like receptor family, pyrin domain containing 3
Th	T helper
TNF	Tumor necrosis factor
UC	Ulcerative colitis
PSC	Primary sclerosing cholangitis
